# Mechanisms and Uses of Dietary Therapy as a Treatment for Epilepsy: A Review

**DOI:** 10.1177/2164956119874784

**Published:** 2019-09-11

**Authors:** Fatemeh Sadeghifar, Vanessa Baute Penry

**Affiliations:** 1 Wake Forest School of Medicine, Winston-Salem, North Carolina; 2 Department of Neurology, Wake Forest School of Medicine, Winston-Salem, North Carolina

**Keywords:** epilepsy, ketogenic diet, ketosis, modified Atkins diet, low glycemic index

## Abstract

One-third of patients with epilepsy do not respond to antiepileptic drugs and may seek complementary and alternative treatment modalities. Dietary therapies such as the ketogenic diet (KD), the modified Atkins, the medium-chain triglyceride, and the low glycemic index diet have been successfully implemented in some forms of epilepsy and are growing in utilization. The KD is a high-fat, low-protein, low-carbohydrate diet that has been used for various conditions for over a century. Insights into the mechanism of action of these diets may provide more targeted interventions for patients with epilepsy. Knowledge of these mechanisms is growing and includes neuroprotective effects on oxidative stress, neuroinflammation, potassium channels in the brain, and mitochondrial function.

## Introduction

Epilepsy affects nearly 50 million people worldwide and costs about $15.5 billion annually in health-care utilization and lost productivity. Antiseizure drugs (ASDs) and surgical interventions have been the mainstays of treatment for epilepsy. However, despite more than 20 different available ASDs, approximately one-third of patients are drug-resistant and continue to experience seizures after adequate trials with 2 or more appropriate medications.^1,2^ Furthermore, side effects and the chronic reliance on ASD polytherapy make the availability of other treatments necessary.

Up to 44% of patients with epilepsy seek complementary and alternative medical therapies to ameliorate seizure burden and improve quality of life.^3^ Notably, the ketogenic diet (KD), a high-fat, low-protein, low-carbohydrate diet, has been utilized clinically as a method for reducing seizure frequency for almost 100 years and has been established as an effective treatment.^4^ The diet has been shown to lead to more than 50% seizure reduction in 36% to 85% of patients and to positively affect cognition.^5^ The efficacy of the KD in terms of seizure reduction is, in most cases, apparent within 2 to 3 months and has been observed in multiple epilepsy syndromes including infantile spasms,^6^ Rett syndrome,^7^ Dravet syndrome,^8^ and GLUT1 deficiency.^9^ The cognitive benefits of short-term (up to 3 months) and long-term KD treatment are promising and result in improved attention, alertness, and adaptability. It is not clear, however, whether this cognitive effect continues upon the discontinuation of the KD.^5^ Despite these positive outcomes, the traditional KD is not without a significant side effect profile including gastrointestinal disturbances, micronutrient deficiencies, hypertriglyceridemia, hyperlipidemia, hypoglycemia, and ketoacidosis, among others.^10,11^ Specifically, gastrointestinal side effects include constipation, diarrhea, nausea, and vomiting, which can improve with minor adjustments.^11^ As such, the implementation of KD is most successful under the guidance of a trained dietitian and neurologist at an epilepsy center.

While mainly studied in pediatric patients, there is evidence that adults also benefit from the KD and its variants. Although seizure reduction is greater with the classic KD (cKD), the modified Atkins diet (MAD) has higher compliance and is more commonly offered to adults due to ease of use.^5,12^ MAD mimics some aspects of the KD while allowing for more incorporation of proteins, fluids, and calories.^13^ In patients remaining on the MAD for at least 6 months, rates of seizure reduction are similar to those reported for long-term KD. In a recent prospective study of patients with drug-resistant epilepsy who began MAD, 44% remained on the diet throughout the study period.^14^ Of those on the diet, 17% had greater than 50% reduction in seizures and 22% became seizure-free.^14^

The medium-chain triglyceride (MCT) diet, a variation of the KD, includes MCTs which are more ketogenic than long-chain triglycerides.^15^ The MCT diet incorporates coconut products such as coconut milk and allows for more carbohydrate and proteins compared to the classical KD. In theory, the production of more ketones coupled with a wider variety of food options would make this the better tolerated dietary therapy. However, a randomized controlled trial of the cKD and the MCT did not show any significant differences in efficacy and tolerability between the 2 diets, although more studies are underway to further compare differences in the 2 diets.^16^

Finally, there has been a recent interest in whether a low glycemic index diet would affect seizure frequency given its role in other medical conditions such as diabetes and heart disease.^17,18^ The diet restricts carbohydrates to 40 to 60 grams per day and is easily implemented. In a retrospective review on 74 pediatric patients who were initiated on the low glycemic index treatment, greater than 50% reduction from baseline seizure frequency was observed in 66% of the population with follow-up at 12 months.^19^

## Mechanisms of Action of Dietary Therapy

**Figure 1.**
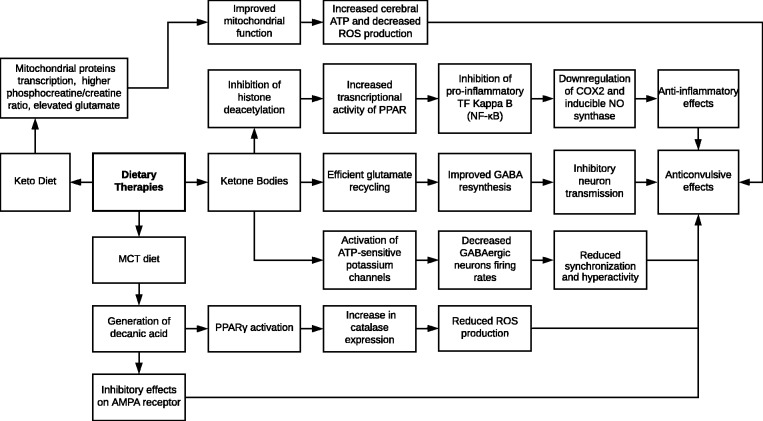
Neuroprotective effects of dietary therapies. GABA, γ-aminobutyric acid; MCT, medium-chain triglyceride; NF-κB, nuclear factor kappa B; PPAR, peroxisome proliferator-activated receptors; ROS, reactive oxygen species.

The mechanisms through which the aforementioned dietary therapies reduce seizure frequency and cortical hyperexcitability are not completely elucidated. However, some theories attribute the anticonvulsant properties of these diets to elevated ketone bodies—one of the byproducts of fatty acid oxidation. Other mechanisms involve changes in gene expression and alterations in mitochondrial function. Figure 1 demonstrates some of the proposed mechanisms through which dietary therapies confer anticonvulsant properties.

### Glutamate Recycling

**Figure 2.**
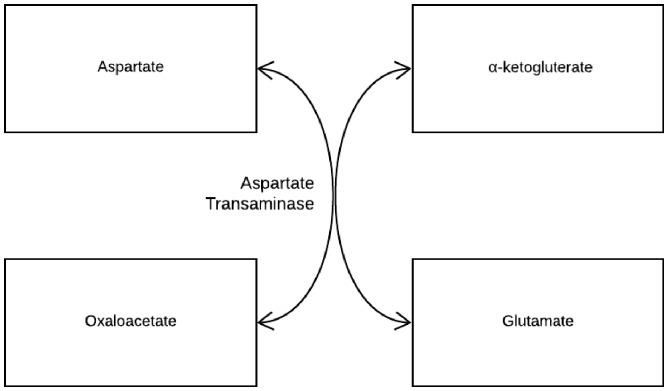
Aspartate transaminase catalyzes the interconversion of aspartate and α-ketoglutarate to oxaloacetate and glutamate.

One of the mechanisms through which ketone bodies can lead to seizure reduction in epilepsy patients is through more efficient glutamate recycling (Figure 1). Metabolism of acetyl-CoA generated from a high-fat diet requires the consumption of oxaloacetate in the Krebs cycle.^20^ Reduced availability of oxaloacetate along with increased availability of α-ketoglutarate leads to low aspartate levels and high glutamate levels (see Figure 2). This higher availability of glutamate allows glutamic acid decarboxylase to produce more γ-aminobutyric acid (GABA), an inhibitory neurotransmitter and an important antiseizure agent.^20,21^ In a study examining the effects of amino acid levels in the cerebrospinal fluid in children with epilepsy, GABA levels were raised, and they were found to be higher in responders (>50% seizure reduction) than in nonresponders during implementation of the diet.^22^

### ATP-Sensitive Potassium Channels (K_ATP_)

Ketone bodies may reduce neuronal excitability by opening ATP-sensitive potassium channels (K_ATP_). K_ATP_s are activated when intracellular ATP levels generated via glycolysis decline. Subsequently, the activation of K_ATP_ slows neuronal firing rates of GABAergic neurons in the substantia nigra pars reticulata (SNpr), which contains a high density of K_ATP_.^22,23^ The SNpr is thought to be a “seizure gate” that regulates seizure threshold, as it participates in neuronal networks that produce synchronization and hyperactivity.^24,25^

### Peroxisome Proliferator-Activated Receptors

Ketone bodies promote histone hyperacetylation by increasing acetyl-CoA, a substrate for histone acetyltransferases, and directly inhibiting histone deacetylases.^23^ This acetylation pattern increases the transcriptional activity of peroxisome proliferator-activated receptors (PPAR) and upregulates endogenous antioxidants genes,^26^ resulting in anti-inflammatory effects. PPARα inhibits pro-inflammatory transcription factor nuclear factor kappa B, which leads to the downregulation in the expression of cyclooxygenase-2 and inducible nitric oxide synthase, both of which are involved in the inflammatory response.^11^

PPARγ is activated by decanoic acid (a component of the MCT) found in the MCT diet.^27^ PPARγ increases catalase expression, preventing the formation of reactive oxygen species (ROS) and subsequently diminishing oxidative damage, which confers neuroprotective effects and contributes to the antiseizure properties of the KD.^27^

Besides activating PPARγ, decanoic acid directly inhibits excitatory ionotropic glutamate AMPA receptor by acting as a noncompetitive antagonist.^28^ AMPA receptors are present in areas of the brain relevant to epilepsy including the cerebral cortex and hippocampus. AMPA receptor antagonists have been shown to have a broad spectrum of anticonvulsant activity in both in vivo and in vitro epilepsy models.^28^

### Mitochondrial Function

Mitochondrial dysfunction is involved in the pathogenesis of neurological diseases through several mechanisms. The KD has been shown to improve mitochondrial function via upregulation of transcripts encoding mitochondrial proteins and a higher phosphocreatine/creatine ratio along with elevated glutamate levels in KD-fed animals.^29^ The dysfunction of complex I in the oxidative phosphorylation pathway can lead to decreased ATP production and increased ROS production, which contributes to cell death.^30^ In aspartate-glutamate carrier (AGC1) deficiency, the shuttling of aspartate from mitochondria to cytosol is impaired and indirectly leads to the transfer of nicotinamide adenine dinucleotide-reducing equivalents into mitochondria, resulting in hypotonia and seizures.^31^ Thus, one role of the KD is to improve mitochondrial function by increasing the efficiency of O_2_ consumption, minimizing oxidative stress and thereby dampen the epileptogenic state.

## Conclusion

Overall, dietary modifications as an adjunctive therapy for seizure reduction is becoming more widespread, is well studied, and has strong clinical and experimental support. Drug-resistant patients who maintain dietary adherence may experience significant reduction and possible seizure freedom. A deeper understanding of the mechanistic underpinnings of dietary modifications in the treatment of epilepsy will likely provide more targeted and better tolerated dietary interventions. Future studies are needed to investigate the optimal duration of the KD for various age groups, to mitigate adverse effects, and to explore the use of dietary therapies in other neurological diseases such as Alzheimer’s disease and neuro-oncology.
